# The Prevalence of Virulence Determinants and Antibiotic Resistance Patterns in Methicillin—Resistant *Staphylococcus aureus* in a Nursing Home in Poland

**DOI:** 10.3390/pathogens10040427

**Published:** 2021-04-03

**Authors:** Martyna Kasela, Agnieszka Grzegorczyk, Bożena Nowakowicz-Dębek, Anna Malm

**Affiliations:** 1 Department of Pharmaceutical Microbiology, Medical University of Lublin, 20-093 Lublin, Poland; agnieszka.grzegorczyk@umlub.pl (A.G.); anna.malm@umlub.pl (A.M.); 2 Department of Animal Hygiene and Environmental Hazards, University of Life Sciences in Lublin, 20-950 Lublin, Poland; bozena.nowakowicz@up.lublin.pl

**Keywords:** *Staphylococcus aureus*, MRSA, nursing home, adhesins, toxins, virulence, MLST, genotyping

## Abstract

Nursing homes (NH) contribute to the regional spread of methicillin-resistant *Staphylococcus aureus* (MRSA). Moreover, residents are vulnerable to the colonization and subsequent infection of MRSA etiology. We aimed at investigating the molecular and phenotypic characteristics of 21 MRSA collected from the residents and personnel in an NH (Lublin, Poland) during 2018. All MRSA were screened for 20 genes encoding virulence determinants (*sea*-*see*, *eta*, *etb*, *tst*, *lukS-F-PV*, *eno*, *cna*, *ebpS*, *fib*, *bbp*, *fnbA*, *fnbB*, *icaADBC*) and for resistance to 18 antimicrobials. To establish the relatedness and clonal complexes of MRSA in NH we applied multiple-locus variable-number tandem-repeat fingerprinting (MLVF), pulse field gel electrophoresis (PFGE), multilocus sequence typing (MLST) and staphylococcal cassette chromosome *mec* (SCC*mec*) typing. We identified four sequence types (ST) among two clonal complexes (CC): ST (CC22) known as EMRSA-15 as well as three novel STs—ST6295 (CC8), ST6293 (CC8) and ST6294. All tested MRSA were negative for *sec*, *eta*, *etb*, *lukS-F-PV*, *bbp* and *ebpS* genes. The most prevalent gene encoding toxin was *sed* (52.4%; *n* = 11/21), and adhesins were *eno* and *fnbA* (100%). Only 9.5% (*n* = 2/21) of MRSA were classified as multidrug-resistant. The emergence of novel MRSA with a unique virulence and the presence of epidemic clone EMRSA-15 creates challenges for controlling the spread of MRSA in NH.

## 1. Introduction

*Staphylococcus aureus* is a major human opportunistic pathogen causing a wide variety of infections both in hospital settings and in the community [1]. Since first methicillin-resistant *Staphylococcus aureus* (MRSA) strains were isolated in the United Kingdom in 1961, they have gradually spread around the world and remain a serious concern for modern infectious medicine [2]. Nowadays, two main MRSA subpopulations are listed, i.e., hospital-associated (HA-MRSA) and community-associated (CA-MRSA). While the former are mainly isolated from hospitalized patients, the latter are usually characterized by a higher virulence but lower antimicrobial resistance and often infect people without significant risk factors for the infection development [3,4]. Infections of MRSA etiology cause multiple therapeutic issues, not only because of resistance to β-lactam antibiotics but also growing resistance to other classes of antimicrobial agents currently used [5].

Another feature enhancing MRSA pathogenicity is the production of toxins and adhesion factors. *S. aureus* is able to produce MSCRAMMs (microbial surface components recognizing adhesive matrix molecules), the role of which has been thoroughly documented in pathogenesis studies [6,7]. Adhesins mediate the effective attachment to host tissues, colonization and biofilm formation but are also responsible for escaping the host’s immune system [8,9]. The severity of symptoms from MRSA infection can also be increased by the production of superantigen toxins. These extracellular proteins overstimulate the immune system, which has been a reason for violent and severe symptoms and a worse prediction of the therapeutic outcome in the case of infection [8]. Studies showed that certain virulent clones of MRSA achieved ecological success and spread more efficiently in the environment; thus, the determination of their genotype provides precious information on the actual local epidemiology [10]. While MLVF (multiple-locus variable-number tandem-repeat fingerprinting) and PFGE (pulsed field gel electrophoresis) methods enable us to compare the relationship of MRSA at the highest resolution, the MLST (multilocus sequence typing) method allows the direct comparison of results between studies.

Beside the hospital setting, long-term care facilities may contribute to the regional spread of MRSA. Nasal MRSA colonization is a well-documented risk factor for the development of endogenous infection [11]. Additionally, studies have shown that older age increases the risk of death in patients with bacteremia of MRSA etiology [12]. Older adults, especially with underlying chronic medical conditions, may easily be affected by MRSA. Because of the resistance to almost all β-lactam antibiotics, MRSA strains are in practice considered as multidrug-resistant microorganisms [13], which is often an indirect reason for a prolonged stay and increased economic burden during hospitalization [14]. The prevalence of MRSA differs significantly between nursing homes (NH), which has been shown by many large-scale studies [15]. The local epidemiological situation within certain institutions depends on multiple factors, mainly environmental and sociodemographic conditions that are complex and unique for each NH. Similar protocols aiming at controlling the spread of multidrug-resistant microorganisms and maintaining proper hygienic measures may be applied to each of them. However, to gain a detailed insight into the spread of MRSA in the environment, it is indispensable to conduct a longitudinal investigation along with a molecular characterization of collected MRSA strains.

Our study aimed at determining the molecular characteristics of MRSA clones isolated during a 12-month investigation from the residents and personnel in NH in Poland. We conducted a complex and longitudinal investigation concerning the antimicrobial resistance profile of MRSA and the presence of adhesion and toxin genes involved in the colonization, carriage and infection states. To gain a detailed insight into the intrafacility spread of certain clones of MRSA, we applied three different genotyping methods: multiple-locus variable-number tandem-repeat fingerprinting (MLVF), pulsed field gel electrophoresis (PFGE) and multilocus sequence typing (MLST).

## 2. Results

During a 12-month investigation, 32.8% of swab samples were found to be *S. aureus*-positive (256/780). Among all *S. aureus* strains collected from the participants, 8.2% were identified as MRSA (21/256).

### 2.1. Antimicrobial Resistance

**Figure 1:**
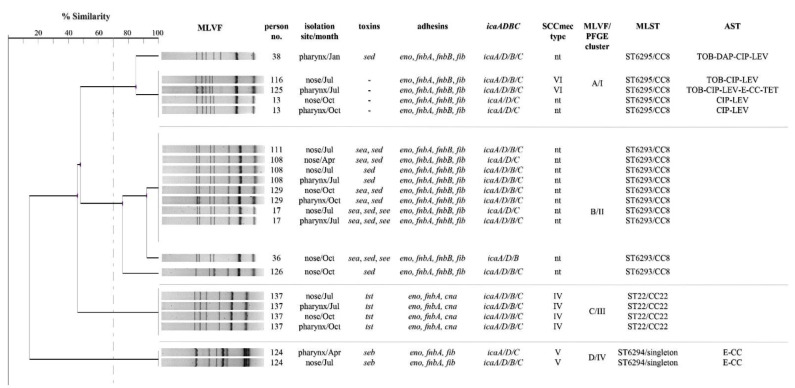
Multiple-Locus Variable-Number Tandem-Repeat Fingerprinting (MLVF) dendrogram of methicillin-resistant *Staphylococcus aureus* strains (cut-off > 70%) showing the molecular and phenotypic characterization results. PFGE—pulsed field gel electrophoresis, MLST—multilocus sequence typing, AST—antimicrobial susceptibility testing, TOB—tobramycin, DAP—daptomycin, CIP—ciprofloxacin, LEV—levofloxacin, E—erythromycin, CC—clindamycin, nt—non-typable. Numbers below 100 have been designated for the residents and those above 100 for personnel members.

All of the tested isolates (*n* = 21) were resistant to penicillin and oxacillin (Figure 1). As much as 66.7% (*n* = 14/21) of the isolated MRSA were resistant only to β-lactam antibiotics (Table 1). Four MRSA (19%) were resistant to one other class of antibiotics: 9.5% (*n* = 2/21) to fluoroquinolones and 9.5% (*n* = 2/21) to macrolide, lincosamides and streptogramins (MLS_B_—constitutive type of resistance phenotype). Multidrug resistance (resistance to at least three groups of antibiotics other than β-lactams) was observed in two MRSA strains (9.5%; *n* = 2/21).

### 2.2. Toxins

All of the tested MRSA strains (*n* = 21) were negative for *sec*, *eta*, *etb* and *lukS-F-PV* genes (Figure 1). Gene *sed* was the most prevalent among the investigated toxins and was harbored by 52.4% (*n* = 11/21) of MRSA strains. Almost half of the isolates possessed only one toxin gene (47.6%; *n* = 10/21). Four isolates possessed two genes at the same time (*sea* and *sed*). The simultaneous presence of three genes was detected in three MRSA (*sea*, *sed* and *see*). Gene encoding for toxic shock syndrome toxin-1 (*tst*) was present in four MRSA isolated from the same personnel member. The internal control gene *femA* was absent in 9.5% (*n* = 2/21) of analyzed MRSA.

### 2.3. Adhesins

Genes *eno* and *fnbA* were the most prevalent and harbored by all of the tested MRSA isolates (Figure 1). As much as 71.4% (*n* = 15/21) of MRSA simultaneously possessed *eno*, *fnbA*, *fnbB* and *fib* genes. Only *bbp* and *ebpS* genes were not detected. In total, 61.9% (*n* = 13/21) of MRSA harbored all genes from the *icaADBC* operon, and the rest of them missed either the *icaB* (23.8%; *n* = 5/21) or *icaC* gene (14.3%; *n* = 3/21).

### 2.4. Genotyping

Among MRSA strains that were typable by SCC*mec* typing (38.1%; *n* = 8/21), four carried SCC*mec* type IV, two SCC*mec* type V and two SCC*mec* type VI. We observed an unusually high percentage of SCC*mec* nontypable MRSA strains (61.9%; *n* = 13/21). Among them, both the *ccr* and *mec* type could not be determined in four strains (30.8%; *n* = 4/13) and the *mec* type could not be determined in two strains harboring *ccr* type 4 (15.4%; *n* = 2/13). In over half of nontypable MRSA strains it was impossible to determine the type of *ccr* (53.8%; *n* = 7/13): except one strain harboring *mec* type C, all had *mec* type B.

The MLST analysis identified four sequence types (ST) and two clonal complexes (CC). We detected three novel sequence types: ST6295 (CC8), ST6293 (CC8) and ST6294 (singleton) as well as typical HA-MRSA ST22-IV (EMRSA-15), which have been deposited in a database (www.pubmlst.org, accessed on 2 April 2021). The highest antibiotic resistance was observed in MRSA from ST6295 (CC8); however, only one strain possessed the single gene responsible for enterotoxin D production. MRSA isolates from ST6293 (CC8) were characterized by a resistance to only β-lactam antibiotics along with the presence of a variable number of enterotoxin genes. The *tst* gene was the only toxin gene detected in ST22 (C22) MRSA. A unique virulence pattern was observed for isolates from ST6294 harboring the *seb* gene as well as exhibiting a constitutive type of resistance phenotype to macrolides, lincosamides and streptogramins. All MRSA strains belonging to CC8 possessed the same adhesin gene profile (*eno*, *fnbA*, *fnbB*, *fib*).

The dendrogram constructed based on the MLVF and PFGE analysis results grouped MRSA into four clusters and seven banding patterns. An identical banding pattern from cluster A/I was shared by MRSA strains isolated from two personnel members and one resident, however at different times of the year. Similarly, three personnel members and one resident were transiently colonized with the same MRSA banding pattern from cluster B/II during the year of study.

## 3. Discussion

As shown by many studies, MRSA prevalence is NHs-dependent. In most of the cases, authors report a low prevalence of MRSA colonization among NH residents and personnel (<10%) [16,17,18,19] or the absence of MRSA [20,21]. Because of the single-swabbing approach applied in most of the studies, a direct comparison of the results is difficult. Considering the emerging data on MRSA in institutions providing specialized care for the elderly, we classified the prevalence of MRSA colonization found in our studies as relatively low (2.7% of positive swab samples).

Contrary to the findings of other studies, we observed a relatively low MRSA resistance rate: as much as 66.7% of all isolated MRSA were only resistant to β-lactams. In Croatian NHs, 75.5% of isolated MRSA were resistant to three or more non-β-lactam classes of antibiotics [17]. Even more alarming results were presented by Moschou et al. (2020) where 97% of MRSA isolated from NHs in Greece were resistant to more than three non-β-lactam classes of antibiotics [22]. Similarly to other authors, we observed that a significant share of MRSA (23.8%) isolated from the elderly and personnel was resistant to fluoroquinolones [23]. The fluoroquinolone resistance eases MRSA selection and limits therapeutic strategies for the treatment of urinary tract or skin infections, thus remaining a serious threat for future medicine [24].

Resistance to agents used for MRSA eradication, e.g., mupirocin and fusidic acid, is being globally observed [15,18,25,26]. In large-scale studies conducted in 60 NHs in Belgium, only 2.7% of MRSA strains were resistant to mupirocin and 3% to fusidic acid [15]. A low prevalence of eradication agent resistance (<10%) was also found in NHs in non-European countries: China [18] and USA [25]. Extremely high resistance rates are rarely being observed but might pose a significant challenge for healthcare, e.g., in Malta, almost all MRSA isolated from healthy individuals were fusidic acid-resistant [26]. We found that none of the MRSA isolated in our study were resistant to fusidic acid and mupirocin, which implies good chances for successful eradication therapy when needed.

A high virulence of MRSA strains colonizing the host might contribute to more severe symptoms of infection. As superantigens, staphylococcal enterotoxins are mainly responsible for food poisoning [27]. Cases of enterocolitis caused by MRSA strains harboring enterotoxins and simultaneously carried in nasal vestibules have been reported [28]. Our studies revealed that gene *sed* encoding enterotoxin D was the most prevalent (52.4%) and that almost half of the MRSA carried only one enterotoxin gene (47.6%), which is consistent with other studies [29]. Along with many other authors [17,23], we found that all MRSA strains isolated from NH were *lukS-F-PV*-negative. Toxic-shock syndrome toxin is a superantigen responsible for toxic shock syndrome, mainly in menstruating women, but also in cases of skin or soft tissue infections, and it remains an important virulence factor in community- and hospital-acquired MRSA infections [30]. The presence of MRSA carrying the *tst* gene might increase the risk of the occurrence of more severe symptoms during endogenous infection. In the multiplex-PCR (polymerase chain reaction) reaction protocol for the detection of toxins proposed by Mehrothra et al. (2000), the *femA* gene was used as an internal control [31]. We observed the absence of this gene in 9.5% of analyzed MRSA (*n* = 2/21). A similar observation has been made by other authors [32,33] that question the application of the *femA* gene as an internal control during the molecular characterization of MRSA strains.

Biofilm formation plays a key role in persistence and the ability to cause infection, e.g., chronic ulcerative lesions of MRSA etiology, independently from high levels of antimicrobial resistance [34]. In our study, only *eno* and *fnbA* genes were carried by all isolated MRSA strains. Some adhesion factors produced by *S. aureus* have a well-documented significance in the development of nasal colonization and carriage [35]. O’Neill et al. (2008) stated that in rare cases *fnbA* and *fnbB* could promote biofilm formation independently from other multifunctional surface proteins [36]. Models investigating the change in the level of expression of genes in *S. aureus* during the shift from colonization to the early bacteremia state revealed that gene *fnbA* was the only one that showed an increase in the level of expression [37]. The *icaADBC* operon encodes the polysaccharide intercellular adhesin (PIA), mediating biofilm formation. Studies proved that, in *S. aureus*, biofilm formation depended not only on the PIA expression levels and genetic background of a certain strain but also on the environmental conditions in vivo. This dependency questions the extrapolation of in vitro results in order to predict biofilm formation in a living organism [38].

The CA-MRSA clones usually carry smaller SCC*mec* elements IV and V than healthcare-associated ones, which carry SCC*mec* type I–III. Nevertheless, there are some exceptions, like epidemic clone EMRSA-15, the predominant HA-MRSA clone in England (CC22/ST22-IV) [39]. Except for the EMRSA-15 clone isolated in our study, we observed the predominance of CA-MRSA. All of the tested MRSA were found to be *pvl*-negative, which undermines the role of the *pvl* gene as a CA-MRSA marker. Despite the fact that most CA-MRSA rarely show resistance to antibiotics other than β-lactams, MRSA isolates in our study that belonged to ST6295 (CC8) demonstrated resistance to multiple classes of antibiotics, including fluoroquinolones, aminoglycosides, lipopetides and tetracyclines, or exhibited a constitutive type of resistance phenotype to macrolides, lincosamides and streptogramins.

Our study has limitations. First, we determined the minimal inhibitory concentration of antibiotics using the Vitek 2 Compact system. Despite the fact that the automated systems produce results in a short period of time, the results are characterized by a lower accuracy when compared to the microbroth dilution method. Second, to establish the pathogenicity of the collected MRSA strains, we detected genes encoding virulence factors; however, the presence of a gene does not mean that it is expressed. Therefore, it is recommended that future studies should also investigate the expression levels of virulence genes.

## 4. Materials and Methods

### 4.1. Collection of Isolates

We collected 21 MRSA strains from the residents and personnel of a NH in Lublin (Poland) during 2018. In total, 101 participants (59 residents and 42 personnel members) were screened for the presence of *S. aureus* in their anterior nares and pharynx. Microbiological sampling was performed quarterly during the year 2018 for almost all of the participants (92/101). The technique of collecting the samples as well as the methods used for identification of *S. aureus* and the detection of methicillin resistance were described in detail previously [40]. The study was approved by the Bioethics Committee at the Medical University of Lublin, Poland (KE 0254/59/2016).

### 4.2. Antimicrobial Susceptibility Testing

All MRSA strains (*n* = 21) were investigated for their phenotypic resistance to ceftaroline, ciprofloxacin, clindamycin, daptomycin, erythromycin, gentamicin, levofloxacin, linezolid, oxacillin, rifampicin, teicoplanin, tetracycline, tigecycline, trimethoprim/sulfamethoxazole and vancomycin with the use of the Vitek 2 Compact system and AST-P644 cards (Biomerieux, Marcy l’Etoile, France) as well as to tobramycin (10 µg), fusidic acid (10 µg) and mupirocin (200 µg) by disc diffusion-method. Additionally, macrolides, lincosamides and streptogramins (MLS_B_) phenotype was detected using the double-disc diffusion test. All discs were obtained from the same manufacturer (Becton Dickinson, New Jersey, NJ, USA). *S. aureus* ATCC 25923 was used as a quality control. All tests were performed and interpreted according to EUCAST recommendations (EUCAST 2018). Isolates were classified as multidrug-resistant according to the criteria proposed by Magiorakos et al. (2012) [13].

### 4.3. Bacterial DNA Isolation

Bacterial DNA was isolated from fresh (24 h) tryptic soy broth (BioRad, Hercules, CA, USA) cultures prepared from single colonies growing on tryptic soy agar (BioRad, Hercules, CA, USA). Isolation of DNA was performed by spin column method according to the manufacturer’s instruction (Genomic Mini, A&A Biotechnology, Gdynia, Poland). Bacterial DNA was stored at 4 °C for further investigation.

### 4.4. Detection of Genes Encoding Toxins and Adhesion Factors

The loci investigated in this study and the used primers are listed in Table 2. A multiplex-PCR protocol described previously [31] was used to detect the following loci responsible for toxin production: *sea* (enterotoxin A), *seb* (enterotoxin B), *sec* (enterotoxin C), *sed* (enterotoxin D), *see* (enterotoxin E), *eta* (exfoliative toxin A), *etb* (exfoliative toxin B) and *tst* (toxic shock syndrome toxin). The *lukS/F-PV* genes encoding the leucocidin Panton–Valentine bicomponent proteins (PVL S/F) were detected using the PCR protocol proposed by McClure et al. (2006) [41]. The presence of genes encoding adhesion factors, *bbp* (bone sialoprotein-binding protein), *cna* (collagen adhesin), *ebpS* (elastin binding protein), *eno* (enolase), *fib* (extracellular fibrinogen-binding protein), *fnbA* (fibronectin binding protein A) and *fnbB* (fibronectin binding protein B), and of genes from the *icaADBC* operon (*icaA*, *icaD*, *icaB*, *icaC*), was determined according to Atshan et al. (2012) [42].

### 4.5. Genotyping of Methicillin-Resistant Staphylococcus aureus

During staphylococcal cassette chromosome *mec* (SCC*mec*) typing, the types of *ccr* and *mec* complex were determined using M-PCR1 and M-PCR2 protocols for SCC*mec* typing designed by Kondo et al. (2007) [43]. The multiple-locus variable-number tandem-repeat fingerprinting (MLVF) of MRSA was conducted using primers proposed elsewhere [44] and a modified PCR program [45] (Whatman, Biometra, Maidstone, UK). The dendrogram was constructed by applying the UPGMA (unweighted pair group method with arithmetic mean) algorithm (BioGene, Polygen, Gliwice, Poland). Pulsed field gel electrophoresis (PFGE) genotyping of MRSA was conducted according to the protocol proposed by Grzegorczyk & Malm (2014) [45] using the CHEF-DR II apparatus and CHEF Bacterial Genomic DNA Plug Kit (BioRad, Hercules, CA, USA). Electrophoresis was conducted using 5–40 s pulses for 21 h with buffer cooling at 13 °C. The obtained electrophoretic profiles were interpreted according to the criteria given by Tenover (1995) [46]. Different band position tolerance settings were compared in both the MLVF and PFGE methods in order to establish the optimal parameter settings. Tolerance values were set at 3%. Proper settings were confirmed by the virulence, antimicrobial resistance and MLST (multilocus sequence typing) data consistency of the clustered MRSA strains, which can be seen in Figure 1. The MLST typing was performed by determining the sequence of seven housekeeping genes (*arcC*, *aroE*, *glpF*, *gmk*, *pta*, *tpiA* and *yqiL*), as described previously [47]. The Sequence type (ST) of all MRSA strains was determined according to the MLST database guidance (www.pubmlst.org, accessed on 2 April 2021). The sequence of the new allele of the *pta* gene has been submitted to the GenBank database (accession No. MW627202).

## 5. Conclusions

Our study investigated the virulence profile of MRSA strains isolated in nursing homes in a 12-month period. We have detected both novel STs of MRSA (ST6295, ST6293 and ST6294) as well as an epidemic EMRSA-15 strain (ST22) among transiently colonized participants, proving that the transmission of MRSA between people is a dynamically changing process. The unique virulence profiles and multidrug resistance among CA-MRSA strains create challenges for controlling the spread of MRSA in NHs. Despite the fact that MRSA were isolated from the colonized individuals, all of them possessed genes encoding for the production of toxins and adhesins increasing the risk of the occurrence of more severe symptoms during endogenous infection.

## Figures and Tables

**Table 1:** Antimicrobial resistance profile of methicillin-resistant *Staphylococcus aureus* (MRSA) in a nursing home.

Category	Number (%) of Resistant MRSA Strains		
	Residents *n* = 6	Personnel *n* = 15	Total *n* = 21
Resistant only to β-lactams	3 (50)	11 (73.4)	14 (66.7)
Resistant to 1 other class	2 (33.3)	2 (13.3)	4 (19)
Resistant to 2 other classes	0	1 (6.7)	1 (4.8)
Resistant to ≥3 other classes	1 (16.7)	1 (6.7)	2 (9.5)

**Table 2:** Target genes, primes sequences and product sizes in PCR assays for the characterization of the collected methicillin-resistant *Staphylococcus aureus* strains.

Gene	Primers	Sequence (5′–3′)	Size (bp)	Reference
*sea*	sea-F	GGTTATCAATGTGCGGGTGG	102	[31]
	sea-R	CGGCACTTTTTTCTCTTCGG		
*seb*	seb-F	GTATGGTGGTGTAACTGAGC	164	
	seb-R	CCAAATAGTGACGAGTTAGG		
*sec*	sec-F	AGATGAAGTAGTTGATGTGTATGG	451	
	sec-R	CACACTTTTAGAATCAACCG		
*sed*	sed-F	CCAATAATAGGAGAAAATAAAAG	278	
	sed-R	ATTGGTATTTTTTTTCGTTC		
*see*	see-F	AGGTTTTTTCACAGGTCATCC	209	
	see-R	CTTTTTTTTCTTCGGTCAATC		
*eta*	eta-F	GCAGGTGTTGATTTAGCATT	93	
	eta-R	AGATGTCCCTATTTTTGCTG		
*etb*	etb-F	ACAAGCAAAAGAATACAGCG	226	
	etb-R	GTTTTTGGCTGCTTCTCTTG		
*tst*	tst-F	ACCCCTGTTCCCTTATCATC	326	
	tst-R	TTTTCAGTATTTGTAACGCC		
*lukS-F-PV*	luk-PV-F	ATCATTAGGTAAAATGTCTGGACATGATCCA	433	[41]
	luk-PV-R	GCATCAAGTGTATTGGATAGCAAAAGC		
*eno*	eno-F	ACGTGCAGCAGCTGACT	301	[42]
	eno-R	CAACAGCATCTTCAGTACCTTC		
*fnbB*	fnbB-F	GTAACAGCTAATGGTCGAATTGATACT	523	
	fnbB-R	CAAGTTCGATAGGAGTACTATGTTC		
*cna*	cna-F	AAAGCGTTGCCTAGTGGAGA	192	
	cna-R	AGTGCCTTCCCAAACCTTTT		
*fnbA*	fnbA-F	CATAAATTGGGAGCAGCATCA	128	
	fnbA-R	ATCAGCAGCTGAATTCCCATT		
*ebpS*	ebpS-F	CATCCAGAACCAATCGAAGAC	180	
	ebpS-R	AGTTACATCATCATGTTTATCTTTTG		
*fib*	fib-F	CTACAACTACAATTGCGTCAACAG	405	
	fib-R	GCTCTTGTAAGACCATTTTCTTCAC		
*bbp*	bbp-F	AACTACATCTAGTACTCAACAACAG	574	
	bbp-R	ATGTGCTTGAATAACACCATCATCT		
*icaA*	icaA-F	ACACTTGCTGGCGCAGTCAA	188	
	icaA-R	TCTGGAACCAACATCCAACA		
*icaD*	icaD-F	ATGGTCAAGCCCAGACAGAG	198	
	icaD-R	AGTATTTTCAATGTTTAAAGCAA		
*icaB*	icaB-F	AGAATCGTGAAGTATAGAAAATT	900	
	icaB-R	TCTAATCTTTTTCATGGAATCCGT		
*icaC*	icaC-F	ATGGGACGGATTCCATGAAAAAGA	1100	
	icaC-R	TAATAAGCATTAATGTTCAATT		
*femA*	femA-F	AAAAAAGCACATAACAAGCG	132	[31]
	femA-R	GATAAAGAAGAAACCAGCAG		
*mecA*	mecA-F	ACTGCTATCCACCCTCAAAC	163	
	mecA-R	CTGGTGAAGTTGTAATCTGG		

## Data Availability

The data presented in this study are available on request from the corresponding author.
